# Supplementary figures and images for: Machine learning for spatial stratification of progressive cardiovascular dysfunction in a murine model of type 2 diabetes mellitus
Source: PLoS One. 2023 May 8;18(5):e0285512. doi: 10.1371/journal.pone.0285512 (PMC10166525; doi:10.1371/journal.pone.0285512)

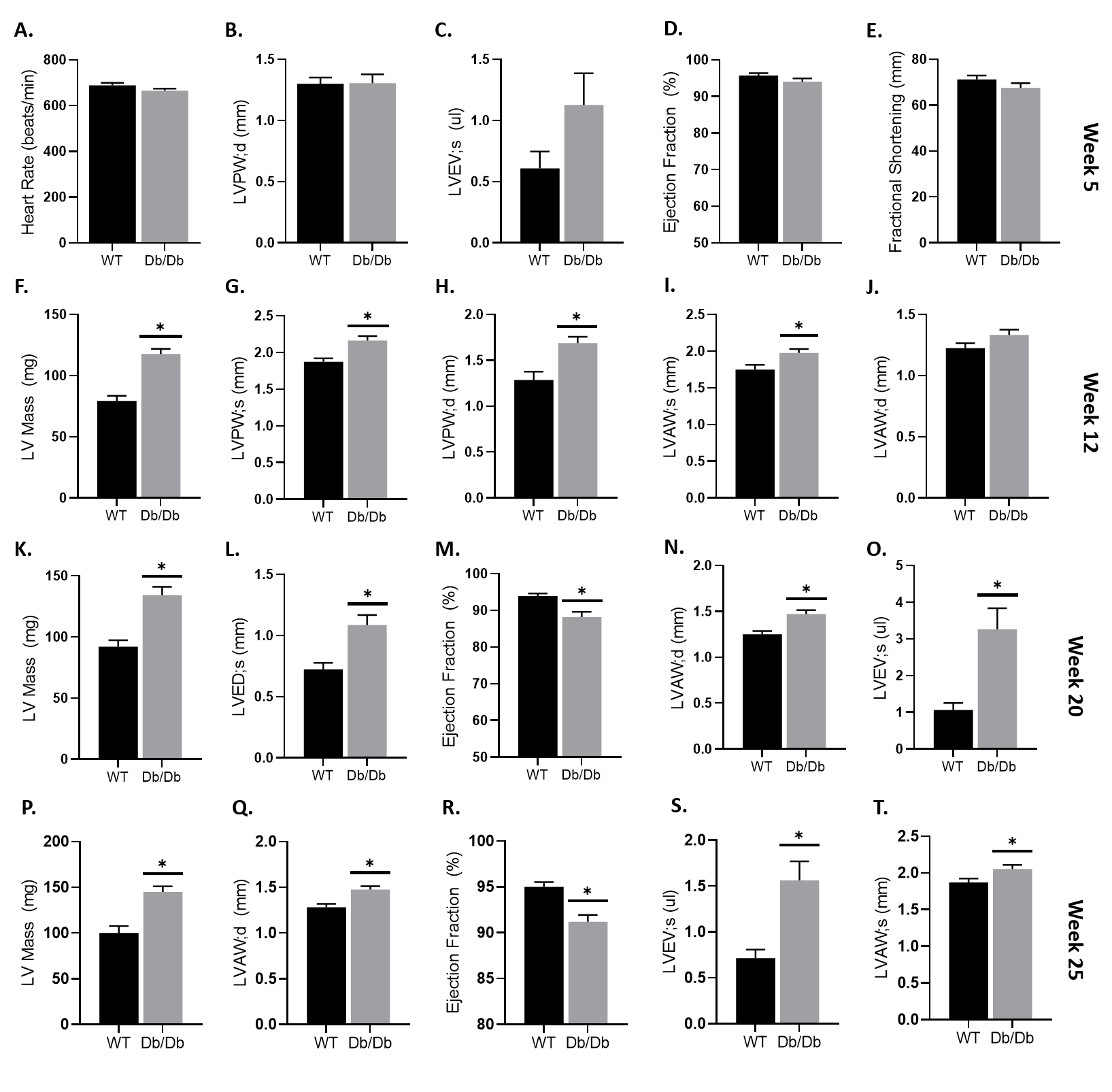

Supplement: S1 Fig — The 5 echocardiography features identified to be most descriptive of cardiac contractile dysfunction selected for 5 weeks; HR, LVPW;d, LVEV;s, EF, FS (A-E), 12 weeks; LV Mass, LVPW;s, LVPW;d, LVAW;s, LVAW;d (F-J), 20 weeks; LV Mass, LVED;s, EF, LVAW;d, LVEV;s (F-G), and 25 weeks; LV Mass, LVAW;d, EF, LVEV;s, LVAW; s (K-O). HR; heart rate, LV; left ventricle, LVPW;d; LV posterior wall diastolic thickness, LVEV;s; LV end-systolic volume, EF; ejection fraction, FS; fractional shortening, LVPW;s; LV posterior wall systolic thickness, LVED;s; LV end-systolic diameter, LVAW;d; LV anterior wall diastolic thickness. “n” is defined as biological replicates. Figure panels are based in 1 independent experiment. WT and Db/Db data were analyzed using a Student’s T-test. “*” Denotes P ≤ 0.05 vs. WT. Values are shown as means ± SEM. (TIF) [file pone.0285512.s001.tif]

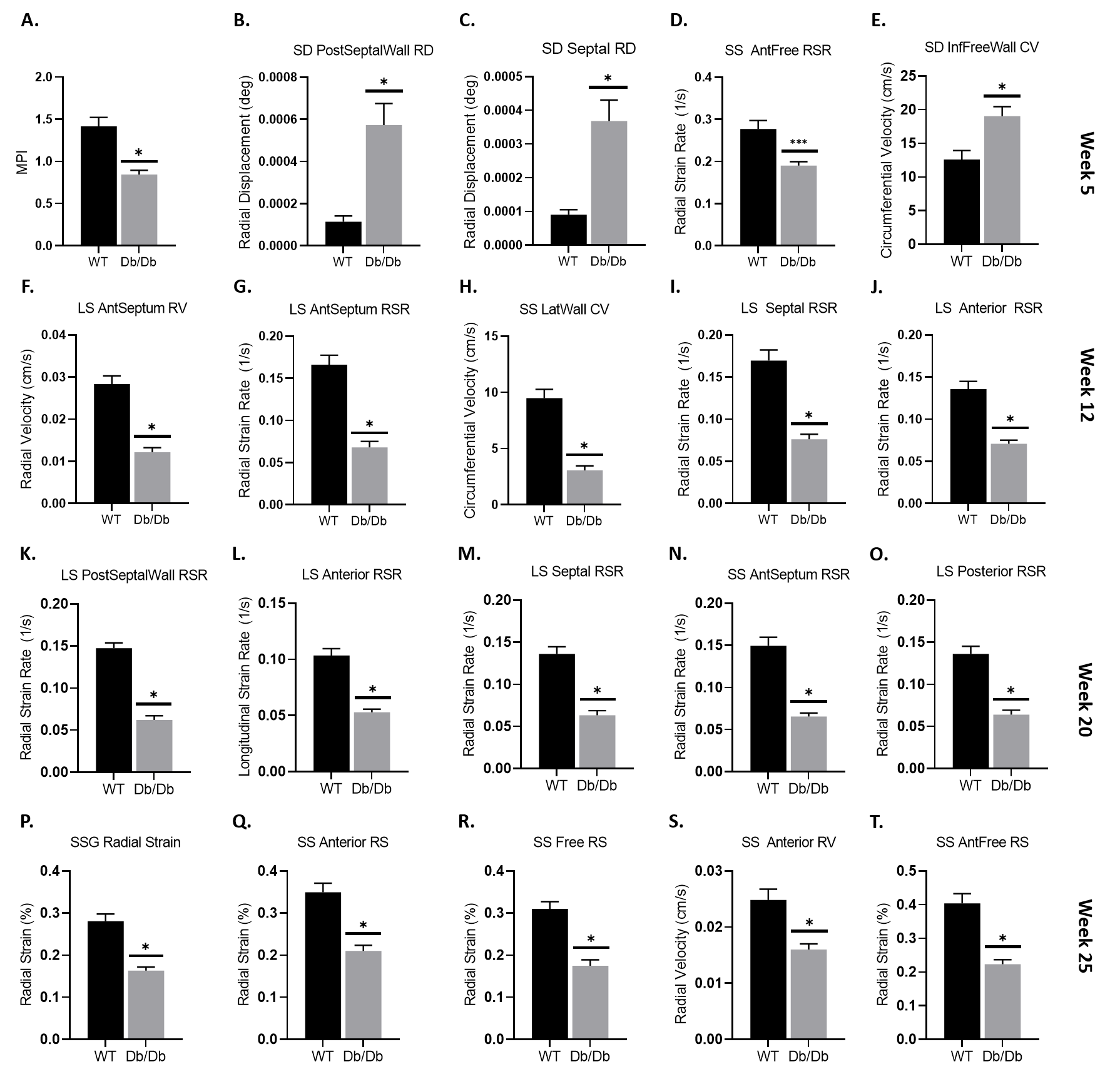

Supplement: S2 Fig — (A-E) The 5 echocardiography features identified to be most descriptive of cardiac contractile dysfunction for 5 weeks, (F-J) 12 weeks, (K-O) 20 weeks, and (P-T) 25 weeks. “n” is defined as biological replicates. Figure panels are based in 1 independent experiment. WT and Db/Db data were analyzed using a Student’s T-test. “*” Denotes P ≤ 0.05 vs. WT. Values are shown as means ± SEM. (TIF) [file pone.0285512.s002.tif]
