# Supplementary material for: Machine learning for spatial stratification of progressive cardiovascular dysfunction in a murine model of type 2 diabetes mellitus
Source: PLoS One. 2023 May 8;18(5):e0285512. doi: 10.1371/journal.pone.0285512 (PMC10166525; doi:10.1371/journal.pone.0285512)
Supplement: S3 Table — The top 50 features were taken and tested as an independent dataset for each timepoint. Training and testing accuracies are reported for both the full and reduced datasets. (DOCX) [file pone.0285512.s005.docx]

| **Timepoint (weeks)** | **Data Subset** | **Training Accuracy** | **Testing Accuracy** | **Number of Features** | **Training Accuracy** | **Std. Deviation** | **Testing Accuracy** | **F-score** |
| --- | --- | --- | --- | --- | --- | --- | --- | --- |
| 5 | Complete | 0.93 | 0.82 | 50 | 1 | 0 | 0.96 | 0.94 |
| 12 | Complete | 0.91 | 0.96 | 50 | 0.92 | 0.05 | 0.98 | 0.97 |
| 20 | Complete | 0.91 | 0.91 | 50 | 0.96 | 0.05 | 0.91 | 0.90 |
| 25 | Complete | 0.97 | 0.89 | 50 | 0.97 | 0.03 | 0.98 | 0.97 |
