# Supplementary material for: Machine learning for spatial stratification of progressive cardiovascular dysfunction in a murine model of type 2 diabetes mellitus
Source: PLoS One. 2023 May 8;18(5):e0285512. doi: 10.1371/journal.pone.0285512 (PMC10166525; doi:10.1371/journal.pone.0285512)
Supplement: S4 Table — Training accuracies and the associated standard deviations, test accuracies, and F-scores are reported. SVM; support vector machine. (DOCX) [file pone.0285512.s006.docx]

| **Timepoint (weeks)** | **Data Subset** | **Training Accuracy** | **Std. Deviation** | **Test Accuracy** | **F-score** |
| --- | --- | --- | --- | --- | --- |
| 5 | AntFree | 0.80 | 0.06 | 0.71 | 0.68 |
|  | AntSeptum | 0.64 | 0.16 | 0.67 | 0.69 |
|  | InfFreeWall | 0.69 | 0.08 | 0.76 | 0.60 |
|  | LatWall | 0.63 | 0.09 | 0.71 | 0.68 |
|  | PostSeptal | 0.68 | 0.12 | 0.73 | 0.71 |
|  | PostWall | 0.54 | 0.07 | 0.49 | 0.48 |
|  | Anterior Free | 0.80 | 0.12 | 0.71 | 0.68 |
|  | Posterior Free | 0.91 | 0.09 | 0.76 | 0.75 |
| 12 | AntFree | 0.83 | 0.11 | 0.84 | 0.83 |
|  | AntSeptum | 0.92 | 0.03 | 0.96 | 0.96 |
|  | InfFreeWall | 0.70 | 0.05 | 0.82 | 0.80 |
|  | LatWall | 0.93 | 0.12 | 0.98 | 0.98 |
|  | PostSeptal | 0.87 | 0.09 | 0.76 | 0.71 |
|  | PostWall | 0.83 | 0.09 | 0.93 | 0.93 |
|  | Anterior Free | 0.93 | 0.02 | 0.98 | 0.98 |
|  | Posterior Free | 0.90 | 0.08 | 0.84 | 0.84 |
| 20 | AntFree | 0.80 | 0.07 | 0.71 | 0.68 |
|  | AntSeptum | 0.80 | 0.08 | 0.96 | 0.96 |
|  | InfFreeWall | 0.87 | 0.03 | 0.71 | 0.64 |
|  | LatWall | 0.88 | 0.07 | 0.93 | 0.93 |
|  | PostSeptal | 0.90 | 0.06 | 0.89 | 0.90 |
|  | PostWall | 0.79 | 0.10 | 0.89 | 0.85 |
|  | Anterior Free | 0.86 | 0.03 | 0.87 | 0.81 |
|  | Posterior Free | 0.77 | 0.11 | 0.78 | 0.79 |
| 25 | AntFree | 0.90 | 0.05 | 0.96 | 0.94 |
|  | AntSeptum | 0.83 | 0.04 | 0.93 | 0.91 |
|  | InfFreeWall | 0.76 | 0.10 | 0.78 | 0.68 |
|  | LatWall | 0.90 | 0.06 | 0.93 | 0.95 |
|  | PostSeptal | 0.82 | 0.06 | 0.60 | 0.59 |
|  | PostWall | 0.73 | 0.07 | 0.60 | 0.51 |
|  | Anterior Free | 0.92 | 0.05 | 0.95 | 0.95 |
|  | Posterior Free | 0.80 | 0.09 | 0.91 | 0.89 |
